# Exploring the Spectrum of Long Non-Coding RNA CARMN in Physiological and Pathological Contexts

**DOI:** 10.3390/biom14080954

**Published:** 2024-08-06

**Authors:** Hui Li, Chuannan Sun, Bin Luo, Chuzhi Zhan, Weitao Li, Lu Deng, Kang Kang, Deming Gou

**Affiliations:** 1 Department of Biochemistry and Molecular Biology, Shenzhen University Medical School, Shenzhen 518060, China; 2200243031@email.szu.edu.cn (H.L.); 2100243041@email.szu.edu.cn (C.S.); 2021225090@email.szu.edu.cn (B.L.); 2021226022@email.szu.edu.cn (C.Z.); 2019221078@email.szu.edu.cn (W.L.); 2 Shenzhen Key Laboratory of Microbial Genetic Engineering, Vascular Disease Research Center, College of Life Sciences and Oceanography, Guangdong Provincial Key Laboratory of Regional Immunity and Disease, Carson International Cancer Center, School of Medicine, Shenzhen University, Shenzhen 518060, China; 2021300051@email.szu.edu.cn

**Keywords:** long non-coding RNA, CARMN, MIR143HG, miR-143, miR-145, cardiovascular diseases, cancer

## Abstract

Cardiac mesoderm enhancer-associated non-coding RNA (CARMN), an evolutionarily conserved long non-coding RNA (lncRNA), serves as the host gene for the miR143/145 cluster. It plays a crucial role in cardiovascular cell differentiation and the maintenance of vascular smooth muscle cell (VSMC) homeostasis, which are vital for normal physiological processes. Specifically, CARMN is associated with the pathological progression of cardiovascular diseases such as atherosclerosis, abdominal aortic aneurysm, and chronic heart failure. Moreover, it acts as a tumor suppressor in various cancers, including hepatocellular carcinoma, bladder cancer, and breast cancer, highlighting its potential as a beneficial biomarker and therapeutic target. This review provides a detailed examination of the roles of CARMN, its evolutionary conservation, expression patterns, and regulatory mechanisms. It also outlines its significant implications in the diagnosis, prognosis, and treatment of these diseases, underscoring the need for further translational research to exploit its clinical potential.

## 1. Introduction

Long non-coding RNAs (lncRNAs) are RNA transcripts that exceed 200 nucleotides in length and lack a discernible protein-coding capacity [1]. Unlike protein-coding genes, which are often evolutionarily conserved for their protein sequences, lncRNA sequences show conservation primarily in their secondary structure and promoter regions [2]. LncRNAs are categorized into five types based on their genomic proximity to protein-coding genes: sense, antisense, bidirectional, intronic, and intergenic. Initially, lncRNAs were dismissed as mere transcriptional noise resulting from RNA polymerase II activity, lacking any functional coding capacity [2]. However, an increasing number of studies have since uncovered that lncRNAs play crucial regulatory roles at various levels of gene expression, including the chromatin remodeling, transcriptional, post-transcriptional, and translational stages. Through diverse mechanisms—serving as signals [3], decoys [4], guides [5], and scaffolds [6]—lncRNAs are integral to key cellular processes such as proliferation, differentiation, stress response, metabolism, and apoptosis, highlighting their significance in cellular biology and as potential therapeutic targets.

CARMN, also known as miR143HG, CARMEN, or NCR143/145, functions as a lncRNA that is intricately associated with cardiac mesoderm enhancers. It plays a pivotal role in a wide array of physiological and pathological processes, acting as a vital regulator or co-factor [7]. Predominantly located in the nucleus across a variety of organs and cell lines, CARMN exhibits notably high expression in cardiac precursor cells (CPCs) during mouse embryonic development. In this setting, it profoundly influences the proliferation and differentiation of cardiovascular cells [8]. Distinctively, CARMN is also expressed in adult vascular smooth muscle cells (VSMCs), where it acts as a critical regulator of VSMC phenotypic stability and transformation [9]. An increasing body of research has highlighted the involvement of CARMN in the initiation and progression of cardiovascular diseases and tumors. This review aims to thoroughly encapsulate the physiological and pathophysiological roles of CARMN, delving into its structure, evolutionary conservation, and mechanisms regulating its expression. Furthermore, we discuss its promising diagnostic and therapeutic applications, which could provide groundbreaking insights and strategies for clinical advancements in managing cardiovascular diseases and cancer.

## 2. Discovery and Genomic Structure of CARMN

CARMN, a super enhancer-associated long non-coding RNA, was initially identified during the profiling of the lncRNA transcriptome in human cardiac precursor cells (CPCs) [10]. Its name, either CARMN or CARMEN, serves as an acronym for (CAR)diac (M)esoderm (E)nhancer-associated (N)on-coding RNA, highlighting its significant involvement in cardiac biology. CARMN is characterized by well-defined transcript boundaries and a non-canonical polyadenylation sequence near its termination site, suggesting sophisticated regulatory mechanisms at play.

**Figure 1:**
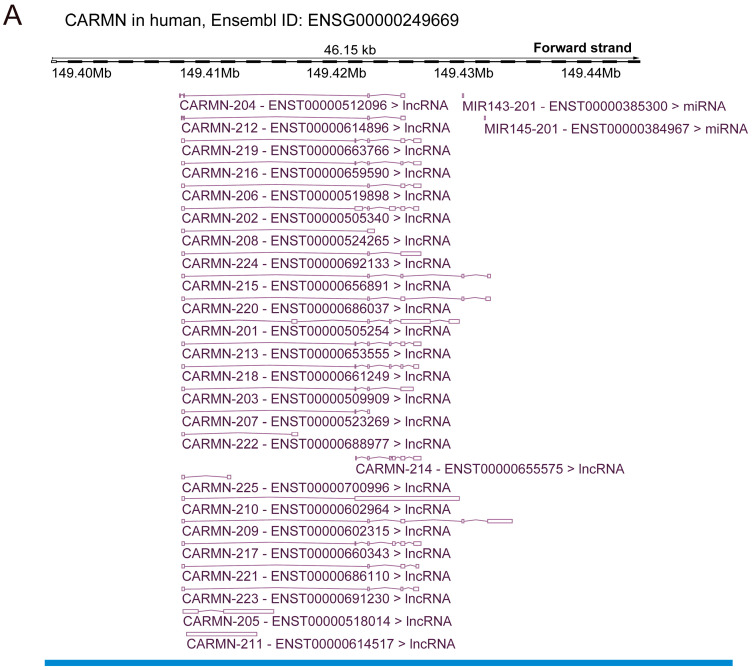

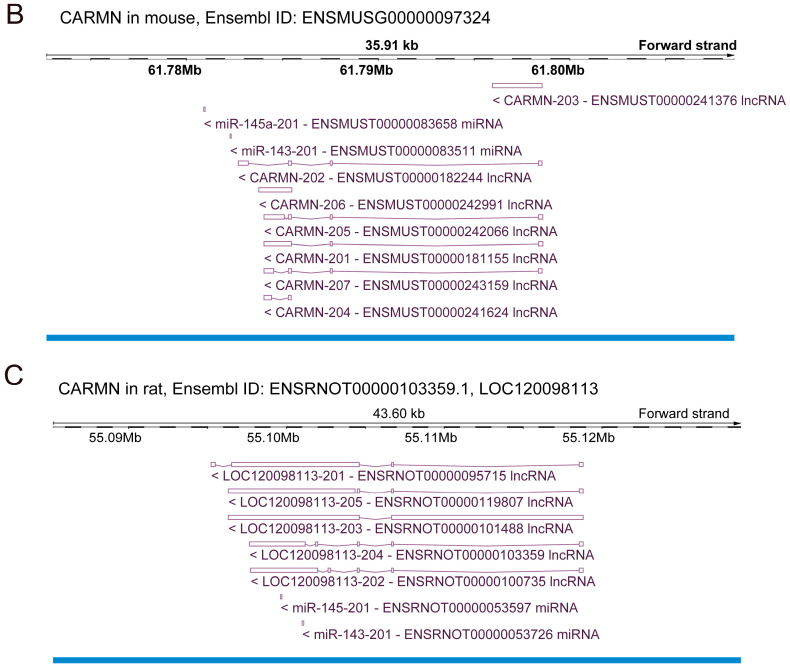
A schematic representation of the CARMN genomic loci in humans, mice, and rats. (**A**,**B**) The human CARMN locus encodes 25 isoforms (**A**), while the mouse counterpart produces 7 isoforms, according to the Ensembl database (**B**). (**C**) LncRNA LOC120098113, co-located with miR-143/145, is suggested to be the unannotated CARMN gene in rats, as indicated in the Ensembl database, yielding 5 isoforms.

Extensive analysis using the Ensembl database has revealed that the human CARMN gene (Ensembl ID: ENSG00000249669) is capable of producing at least twenty-five distinct isoforms, whereas its mouse counterpart (Ensembl ID: ENSMUSG00000097324) generates seven isoforms. In rats, while CARMN is annotated in the NCBI Gene database (accession: NR_184396), it remains unannotated in the Ensembl database. However, detailed comparative analyses have shown that the rat lncRNA LOC120098113 (Ensembl ID: ENSRNOT00000103359.1), producing at least five transcripts, shares high homology with the CARMN gene NR_184396 annotated at NCBI. Additionally, LOC120098113 is co-located with miR-143/145, solidifying its identity as the rat equivalent of the unannotated CARMN gene (Figure 1).

The initial studies suggested that the biogenesis of CARMN was independent of its neighboring microRNAs (miRNAs), miR-143/145, suggesting the occurrence of a distinct regulatory pathway. However, the dynamic interplay between CARMN and miR-143/145 has increasingly attracted scientific interest and is poised to offer new insights into the regulatory networks of non-coding RNAs, which will be explored in depth in subsequent sections of this review.

## 3. Physiological Functions of CARMN

Within the intricate landscape of cellular processes and differentiation, CARMN emerges as a pivotal regulatory molecule. This section comprehensively delineates the multifaceted physiological roles of CARMN, emphasizing its critical significance in determining cell fate, maintaining smooth muscle cell (SMC) homeostasis and phenotypes, and regulating both stem cell renewal and the maintenance of stemness characteristics.

### 3.1. Regulating Cardiomyocyte Differentiation

**Figure 2:**
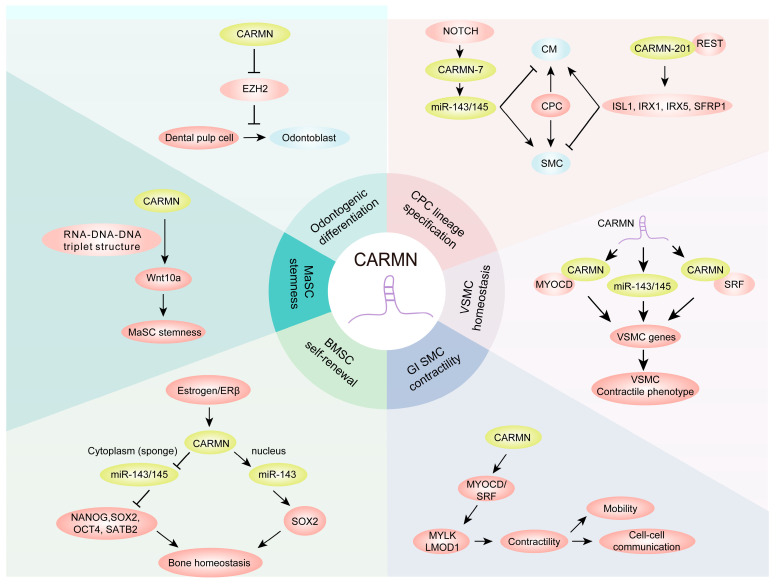
Physiological roles of CARMN. CARMN plays a pivotal role in the regulation of CPC specification, maintenance of homeostasis in BMSC and MaSC, and modulation of SMC and DPC differentiation. CM: cardiomyocyte; VSMC: vascular smooth muscle cell; MaSC: mammary gland stem cell; DPC: dental pulp cell.

Plaisance et al. identified CARMN as a pivotal regulator of cardiac specification in human CPCs [8]. Although these adult human CPCs primarily generate SMCs, their differentiation into cardiomyocytes can be effectively induced through the NOTCH signaling pathway (Figure 2). Inhibition of NOTCH signaling results in the downregulation of miR-143/145, which are crucial for maintaining smooth muscle differentiation. Notably, CARMN, particularly its isoform CARMN-7 (referred to as CARMEN-7 in the article), has a significant impact on miR-143/145 expression, thereby steering the fate of adult CPCs towards either cardiomyocytes or SMCs. Moreover, they demonstrated that a key isoform, CARMN-201 (also known as CARMEN-201 in the article, formally CARMEN-7), is critical for SMC lineage commitment [11]. CARMN-201 features an alternatively spliced exon with a mammalian-wide interspersed repeat (MIR) element, a type of short interspersed nuclear element (SINE). This MIR element in CARMN-201 interacts with the transcriptional repressor REST (RE1-silencing transcription factor), leading to the inhibition of several essential cardiogenic genes, including ISL1, IRX1, IRX5, and SFRP1, thereby influencing cardiac cell fate determination. These insights shed light on the complex molecular mechanisms that govern heart cell differentiation and point to potential novel targets for heart regeneration and repair therapies.

### 3.2. Maintaining VSMC Homeostasis

VSMCs are critical to maintaining the physiological functions of blood vessels. CARMN, which is highly expressed in VSMCs, is activated by TGF-β1 through a Smad2/3-dependent pathway [12,13]. The loss of CARMN in human coronary arterial smooth muscle cells (hCASMCs) results in significant transcriptomic changes which adversely affect VSMC proliferation, migration, inflammation, lipid metabolism, and the process of dedifferentiation. Furthermore, CARMN plays an essential role in the transition of VSMCs from a contractile to a synthetic phenotype [12], a shift that is vital for vascular remodeling and repair. Its primary mechanism involves interaction with myocardin (MYOCD), a key transcriptional cofactor for smooth muscle cell differentiation, enhancing its regulatory impact on smooth muscle-specific gene promoters. Moreover, CARMN has been identified as a crucial regulator of VSMC plasticity, interacting with serum response factor (SRF) to amplify its targeting of gene promoters involved in smooth muscle function [13]. Remarkably, CARMN also modulates VSMC phenotypes independently of the miR-143/145 cluster [12,13], demonstrating its unique role in vascular function. These findings highlight the central role of CARMN in vascular biology and support the potential of CARMN modulation as a therapeutic strategy for treating vascular diseases characterized by abnormal VSMC proliferation and differentiation.

### 3.3. Modulating GI SMC Phenotypes

Visceral SMCs are critical for gastrointestinal (GI) tract functionality, playing a pivotal role in regulating GI motility. He et al. discovered that CARMN is highly expressed in the GI SMCs of both humans and mice, indicating its significant biological relevance [14]. CARMN deficiency notably affects GI SMC phenotypes, primarily through the upregulation of extracellular matrix genes and the downregulation of smooth muscle contractile genes. Mechanistically, CARMN interacts with the SRF/MYOCD complex, significantly enhancing the expression of key SMC genes, including MYLK and LMOD1, in human colonic SMCs (HuCoSMCs). This interaction underscores CARMN’s role not only in maintaining SMC contractility but also in the broader context of cellular function within the GI tract. Overall, this study highlights the critical role of the lncRNA CARMN in modulating GI SMC phenotypic switching, contractility, and motility and illuminates its potential implications in managing and understanding GI disorders.

### 3.4. Regulating BMSC Self-Renewal

Bone mesenchymal stem cells (BMSCs) play a crucial role in maintaining bone cell homeostasis, primarily through their capabilities in self-renewal and differentiation. The lncRNA CARMN has been shown to interact with miR-143/145, forming complexes that are vital for regulating BMSC functions [15]. In the cytoplasm, CARMN binds to miR-143/145, acting as a competing endogenous RNA (ceRNA). This interaction significantly influences the translation of key pluripotency genes, including NANOG, SOX2, OCT4, and SATB2, which are essential for maintaining stem cell properties. Furthermore, within the nucleus, CARMN collaborates with miR-143 to facilitate the co-activation of SOX2 transcription, a process that involves the remodeling of promoter chromatin to profoundly influence gene expression at the transcriptional level. This study not only highlights the significant role of CARMN in the pathology of estrogen-deficient bone loss but also suggests that strategically targeting the CARMN-miR-143/145 axis could represent a novel therapeutic approach for osteoporosis, providing new avenues for clinical research and treatment strategies in bone health management.

### 3.5. Sustaining MaSC Stemness

In a pivotal study on mammary gland stem cells (MaSCs), Xu et al. identified CARMN as a lineage-determining gene, prominently expressed in both MaSCs and basal cells [16]. This lncRNA specifically regulates the Wnt signaling ligand Wnt10a by forming a unique RNA-DNA-DNA triplex structure. It leverages the triplex target sites (TTSs) of Wnt10a and the triplex-forming oligonucleotides (TFOs) of CARMN to modulate the pathway. Wnt signaling is crucial for processes such as cell proliferation and the maintenance of stem cell properties and is significantly influenced by CARMN, thus affecting the stemness and self-renewal capabilities of MaSCs. Given these findings, CARMN has emerged as a potential therapeutic target, particularly for conditions affecting mammary gland development and associated disorders, highlighting its importance in regenerative medicine and oncology.

### 3.6. Promoting Odontogenic Differentiation

Odontogenic differentiation, the transition of dental pulp cells (DPCs) into odontoblasts, is crucial for dentin formation during tooth development. CARMN expression is notably higher in odontoblasts than in DPCs of P0 mice, indicating its significant role in odontogenic processes [17]. CARMN facilitates the odontogenic differentiation of DPCs by suppressing EZH2 (enhancer of zeste 2 polycomb repressive complex 2 subunit), a histone methyltransferase. This suppression effectively promotes the formation of mineralized nodules. These findings elucidate the molecular mechanisms underlying dentin-pulp complex regeneration, offering potential therapeutic targets for dental tissue engineering and regenerative dentistry. Further studies are needed to explore the full extent of CARMN’s role in dental tissue development and its applications in clinical settings.

## 4. Pathological Functions of CARMN

The pathological roles of CARMN underscore its pivotal involvement in a wide spectrum of diseases. This section explores the diverse pathological functions of CARMN, emphasizing its crucial role in the development of atherosclerosis, abdominal aortic aneurysm, chronic heart failure, Hirschsprung disease, airway stenosis, and various types of cancer. Such insights reveal the potential of CARMN as a therapeutic target, offering novel pathways to combat these debilitating conditions.

### 4.1. Atherosclerosis

**Figure 3:**
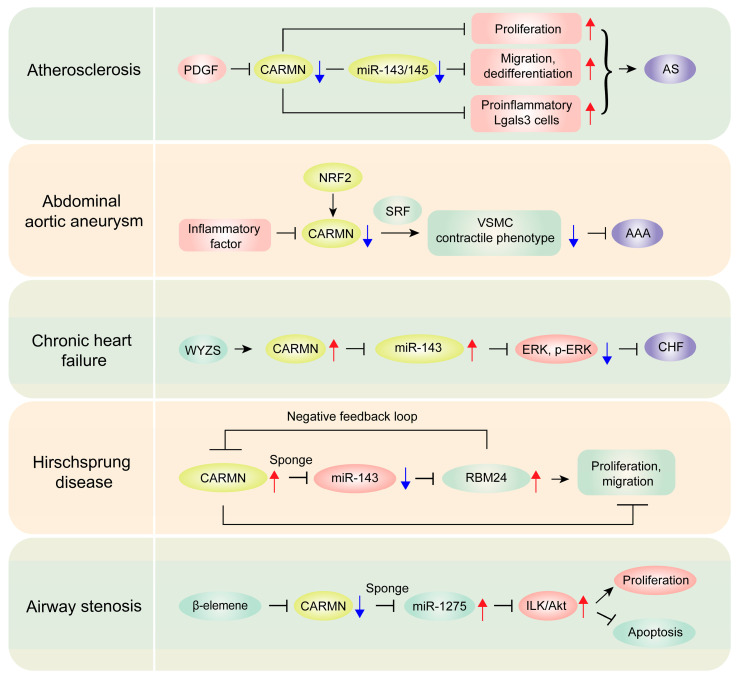
Role of CARMN in modulating molecular pathways across cardiovascular, gastrointestinal, and respiratory diseases. It elucidates the diverse roles of CARMN in influencing pathophysiological processes in atherosclerosis, abdominal aortic aneurysm, chronic heart failure, Hirschsprung disease, and airway stenosis. The specific molecular pathways and interactions implicated in these conditions are detailed. The red arrow signifies upregulation, while the blue arrow denotes downregulation.

Atherosclerosis (AS) is a chronic inflammatory arterial disease, characterized by lipid accumulation and active inflammation [18].VSMCs play a critical role at both the early and late stages of AS. Research has shown that CARMN and its adjacent miR-143/145 cluster are downregulated in advanced versus early atherosclerotic lesions in both humans and mice, highlighting the significant involvement of CARMN in the progression of AS [19] (Table 1). A CRISPR-Cas9 knockout study demonstrated that a deficiency in CARMN leads to exacerbated AS symptoms (Figure 3). The affected knockout mice exhibited not only increased plaque volume and size but also a rise in pro-inflammatory Lgals3 (galectin-3)-expressing cells, alongside altered plaque composition, indicating a more advanced disease state [19]. These observations underscore the extensive regulatory influence of CARMN on VSMC behavior and the overall progression of AS. Intriguingly, Ni et al. reported that knocking down CARMN in low-density lipoprotein (LDL) receptor knockout (LDLR−/−) mice significantly reduced atherosclerotic lesion formation and inhibited VSMC proliferation without impacting apoptosis [13]. These findings not only highlight the critical and complex roles of CARMN in AS development but also suggest its potential as a novel therapeutic target for vascular diseases.

### 4.2. Abdominal Aortic Aneurysm

An abdominal aortic aneurysm (AAA) is a life-threatening dilation of the aorta, currently lacking effective pharmacological treatments [20]. In AAA, a significant reduction in contractile proteins within aortic SMCs correlates strongly with decreased expression of CARMN [21,22]. This downregulation is thought to be driven primarily by the inflammatory environment, particularly through macrophage activity [21]. Research indicates that macrophage-conditioned media can reduce CARMN levels, suggesting that inflammation-mediated modulation of this lncRNA may contribute to AAA pathogenesis by impairing SMC contractility. Additionally, CARMN interacts with SRF, playing a protective role by maintaining the contractile phenotype of VSMCs [22]. Moreover, NRF2, a transcription factor associated with oxidative stress response, has been shown to enhance CARMN transcription by binding to its promoter. This indicates a complex regulatory mechanism that influences VSMC phenotype and the pathology of AAA.

### 4.3. Chronic Heart Failure

Chronic heart failure (HF) is increasingly prevalent, necessitating deeper investigation into its pathophysiological mechanisms [23]. Xu et al. demonstrated that CARMN directly modulates miR-143, significantly impacting the expression and phosphorylation of the ERK5 protein in H9C2 cardiomyocytes in cases of HF [24]. Moreover, the researchers showed that Wenyang Zhenshuai granule, a traditional Chinese medicine formula, mitigates doxorubicin-induced cardiomyocyte injury by regulating the expression of CARMN and miR-143. This evidence strongly suggests the potential of CARMN as a therapeutic target in the treatment of chronic heart failure.

**Table 1:** Functional characterization of CARMN in diseases.

Disease	Expression	Biological Function	Related Genes	Mechanisms	Reference
Atherosclerosis	Down	Dedifferentiation, proliferation, migration	SRF, MYOCD	Host gene, PDGF-BB, TGF-β1/Smad 2/3	[13,19]
Abdominal aortic aneurysm	Down	VSMC phenotype	Il1b, NRF2	SRF	[21,22]
Chronic heart failure	Down	Cardiomyocyte injury	miR-143	Sponge, ERK	[24]
Hirschsprung disease	Up	Proliferation, migration	miR-143, RBM24	Sponge, negative feedback loop	[25]
Airway stenosis	Down	Proliferation, apoptosis	miR-1275	Sponge, ILK/Akt	[26]
Thoracic aortic aneurysms	Down	Biomarker	LUCAT1, MALAT1, and SMILR	N/A	[27]
Hepatocellular carcinoma	Down	Cell motility, cell cycle arrest and proliferation	miR-155, APC, β-catenin, ZEB1, E-cadherin	Sponge, MAPK, Wnt/β-catenin	[28]
Bladder cancer	Down	Proliferation, invasion	miR-1275, AXIN2	Sponge, Wnt/β-catenin	[29]
Breast cancer	Down	Growth, chemotherapy sensitivity, EMT	miR-143-3p, MCM5, MMP2, DHX9	Host gene, DNA replication	[30]
Endometrial carcinoma	Down	Apoptosis	p53, miR-125a	Sponge	[31]
Gastric cancer	Down	Growth	DDX6, miR-143/145	Host gene, RNA degradation	[7]
Cervical cancer	Down	proliferation, migration, invasion, apoptosis	miR-92a-3p, BTG2	Sponge, Wnt/β-catenin	[32]
Glioma	Down	Proliferation	miR-504, p53	Sponge	[33]
Laryngeal squamous cell carcinoma	Down	Migration, invasion	miR-21	DNA methylation	[34]
Colorectal cancer	N/A	Prognosis	Transcription factors	Sponge, focal adhesion, Wnt signaling	[35]
	Down	Proliferation, invasion	P53, ALKBH5, miR-5683, and FGF2	RNA methylation	[36]

N/A: not available; Up: upregulation; Down: downregulation.

### 4.4. Hirschsprung Disease

Hirschsprung disease (HSCR) is a congenital disorder causing intestinal motility issues due to missing enteric nervous system ganglia [37]. Du et al. demonstrated that CARMN regulates cell proliferation and migration in HSCR through a complex interaction with RBM24 and miR-143 [25]. CARMN functions as a molecular sponge for miR-143, indirectly upregulating RBM24, a protective factor. This upregulation, in turn, destabilizes CARMN, establishing a negative feedback loop. This regulatory circuit significantly impacts cell behavior, as the knockdown of CARMN and overexpression of miR-143 result in enhanced cell proliferation and migration. Furthermore, CARMN is frequently elevated in HSCR patient tissues compared to control tissues, suggesting its potential as a predictive marker for the clinical prognosis of HSCR. These findings highlight the importance of CARMN in HSCR pathology and suggest that targeting this lncRNA could provide new therapeutic strategies for the disease.

### 4.5. Airway Stenosis

Airway stenosis is a pathological narrowing of the airways, often resulting from inflammation, fibrosis, or external compression. Research shows that exposure to β-elemene significantly downregulates CARMN, which functions as a competing endogenous RNA (ceRNA) for miR-1275 [26]. This interaction indirectly modulates the integrin-linked kinase (ILK)/Akt signaling pathway. The reduction in CARMN by β-elemene results in the upregulation of miR-1275, which subsequently diminishes ILK/Akt pathway activity. This decreased pathway activity curtails proliferation and enhances apoptosis in airway granulation fibroblasts, effectively mitigating airway stenosis. Consequently, CARMN has emerged as a crucial molecular mediator in the β-elemene-driven therapeutic approach for managing airway stenosis.

### 4.6. Tumors

#### 4.6.1. Hepatocellular Carcinoma

**Figure 4:**
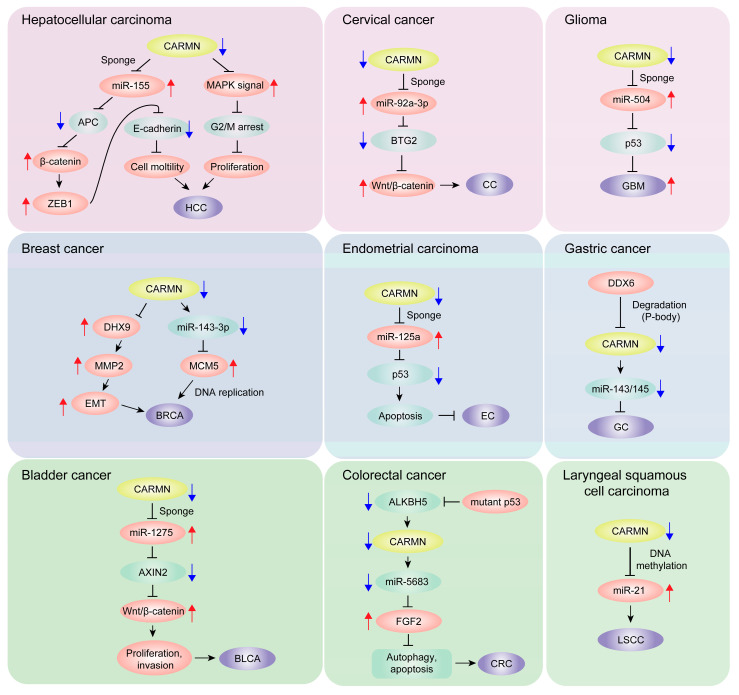
Impact of CARMN on tumor pathophysiology and molecular interactions. The role of CARMN as a tumor suppressor is depicted through its interactions with miRNAs and critical signaling pathways. The figure illustrates how CARMN modulates cancer cell proliferation, migration, and invasion across various tumor types. The red arrow signifies upregulation, while the blue arrow denotes downregulation.

Hepatocellular carcinoma (HCC), the primary liver cancer, arises from chronic liver diseases and presents significant global health challenges [38]. In HCC, significantly downregulated CARMN expression correlates with improved prognoses, serving as an independent prognostic factor for overall survival [28]. Overexpression of CARMN leads to the suppression of miR-155, which in turn targets the adenomatous polyposis coli (APC), a negative regulator of the Wnt/β-catenin signaling pathway (Figure 4). This suppression leads to decreased ZEB1 expression, resulting in enhanced E-cadherin expression and reduced cell motility. Additionally, CARMN interferes with the mitogen-activated protein kinase (MAPK) signaling pathway, influencing both cell cycle arrest and cellular proliferation. Collectively, these insights underscore the potential of CARMN-targeted therapeutic strategies in the management of HCC.

#### 4.6.2. Bladder Cancer

Bladder cancer (BLCA), the second most common urogenital malignancy globally, results in significant morbidity and mortality [39]. In studies of BLCA, CARMN expression is notably lower in cancerous tissues compared to normal tissues [29]. Elevated CARMN levels are associated with improved survival rates in BLCA patients, highlighting its potential as a prognostic marker. Mechanistically, CARMN exerts its effects by inhibiting miR-1275, which targets AXIN2, a crucial negative regulator of the Wnt/β-catenin signaling pathway. This inhibition effectively suppresses BLCA cell proliferation, halts cell cycle progression, and reduces migration and invasion capabilities. Consequently, these interactions position the CARMN/miR-1275/AXIN2/Wnt/β-catenin pathway as a promising therapeutic target for BLCA.

#### 4.6.3. Glioma

Targeting mutant p53 presents a promising therapeutic approach in cancer therapy [40]. In glioblastoma (GBM) tissues, the expression of CARMN is notably reduced, and higher levels of CARMN are associated with enhanced survival rates [33]. CARMN acts by sponging miR-504, which leads to the upregulation of p53 and subsequent inhibition of GBM cell proliferation. This mechanism highlights the critical role of CARMN as a tumor suppressor within GBM. Ultimately, CARMN has emerged as a pivotal factor in GBM management, offering promising pathways for intervention and advancing our comprehension of the molecular mechanisms underlying GBM pathogenesis.

#### 4.6.4. Gastric Cancer

Gastric cancer (GC), a prevalent malignancy, has a poor five-year survival rate under 30% for advanced stages [41]. In GC, a significant reduction in the expression of CARMN and miR-143/145 is observed, underscoring their potential involvement in the onset and progression of the disease [7]. This decline is orchestrated by DEAD-box RNA helicase 6 (DDX6), which suppresses miR-143/145 expression by destabilizing CARMN RNA within cellular processing bodies (P-bodies). These findings suggest that inhibiting DDX6, thereby increasing miR-143/145 levels, could hinder cancer cell proliferation. This evidence points to the DDX6-CARMN-miR-143/145 axis as a promising target for therapeutic intervention in GC treatment.

#### 4.6.5. Laryngeal Squamous Cell Carcinoma

Recent research has increasingly focused on the role of various lncRNAs in laryngeal squamous cell carcinoma (LSCC) [42]. Notably, CARMN is significantly downregulated in LSCC and is associated with poorer patient survival outcomes [34]. Studies have demonstrated that overexpression of CARMN reduces levels of the oncogenic miRNA, miR-21, through mechanisms involving DNA methylation. This reduction leads to a decreased capacity for invasion and migration in LSCC cells. Therefore, CARMN acts as a tumor suppressor by negatively regulating miR-21, thereby curtailing the aggressive behavior of cancer cells. This underscores the therapeutic potential of targeting CARMN in LSCC to modulate miR-21 levels and inhibit tumor progression.

#### 4.6.6. Endometrial Carcinoma

Endometrial cancer (EC), the most prevalent pelvic gynecological malignancy in high-income nations, carries a 2–3% lifetime risk and often recurs post-surgery [43]. In the study conducted by Shi et al., it was found that lower levels of CARMN in patients with EC are correlated with poorer survival outcomes [31]. The research further illustrated that CARMN functions as a tumor suppressor in EC. It achieves this by upregulating the tumor suppressor protein p53 through the sequestration of miR-125a, which consequently enhances apoptosis in cancer cells. This mechanism not only underscores the pivotal role of CARMN in modulating cellular processes but also suggests its potential impact on improving survival outcomes in EC patients.

#### 4.6.7. Cervical Cancer

Cervical cancer (CC), the fourth most common cancer among women worldwide, predominantly arises from human papillomavirus (HPV) infection [44]. In CC tissues and cell lines, significantly lower levels of CARMN have been observed, which correlate with adverse clinical outcomes and disease characteristics [32]. Research indicates that elevated CARMN expression suppresses miR-92a-3p, thereby increasing the expression of the B-cell translocation gene 2 (BTG2). This upregulation of BTG2 results in decreased cell proliferation, migration, and invasion while concurrently promoting apoptosis in CC cells. Moreover, CARMN disrupts the Wnt/β-catenin signaling pathway by modulating the miR-92a-3p/BTG2 axis, highlighting its potential as a therapeutic target for altering the course of CC progression.

#### 4.6.8. Breast Cancer

Breast cancer is the most prevalent cancer among women, characterized by significant molecular heterogeneity [45]. In breast cancer, CARMN is significantly downregulated, and its expression levels are closely associated with patient outcomes, establishing CARMN as a crucial predictive biomarker for this disease [30,46]. Sheng et al. highlighted that CARMN serves as a host gene for miR-143-3p, which targets and suppresses MCM5, a critical gene involved in DNA replication [30]. This suppression of MCM5, mediated by CARMN and miR-143-3p, inhibits DNA replication, leading to reduced tumor growth in triple-negative breast cancer (TNBC). Moreover, this pathway significantly enhances the sensitivity of TNBC cells to cisplatin, offering a potential therapeutic leverage point. Additionally, Liao et al. demonstrated that CARMN impedes the transcription of MMP2 by competitively binding to DHX9, a known transcriptional activator of MMP2. This interference disrupts the epithelial–mesenchymal transition (EMT) process in breast cancer [47], further underscoring the role of CARMN in modulating key cancer progression pathways.

#### 4.6.9. Colorectal Cancer

In colorectal cancer (CRC), CARMN undergoes hypermethylation due to m^6^A modifications, which diminishes its expression and contributes to tumor growth and metastasis [36]. The demethylase ALKBH5 typically protects CARMN by reversing these modifications. However, mutant p53 inhibits ALKBH5, sustaining the methylation of CARMN. Restoring CARMN expression impedes tumor progression, in part by reducing FGF2 via miR-5683, pointing to demethylation strategies as viable therapeutic options. The study conducted by Cai et al. on rectal cancer underscores the crucial role of lncRNA–mRNA interactions, particularly highlighting CARMN as a pivotal node within a rectal cancer-associated network involving MBNL1-AS1 [35]. This network significantly influences cancer pathways like focal adhesion and Wnt signaling. The findings suggest the central regulatory and prognostic significance of CARMN in rectal cancer progression.

## 5. Genetic Variations of CARMN

Recent studies have elucidated the pivotal role of CARMN single nucleotide polymorphisms (SNPs) in influencing disease susceptibility and outcomes among the Chinese population. Studies on alcohol-induced osteonecrosis of the femoral head (ONFH) revealed that genotypes rs13177623 and rs12654195 are linked to a reduced risk [48], underscoring the utility of CARMN variations as biomarkers. Moreover, investigations into esophageal cancer risk have highlighted the protective effects of the TT genotype of rs353299 and a particular haplotype combination of Trs11168100Ars353303Trs353300Crs353299 [49], further demonstrating the broad applicability of CARMN SNPs in disease risk assessment.

Concurrently, specific CARMN polymorphisms, particularly rs11168100 and rs353300, are significantly associated with gastric cancer (GC) risk, with rs11168100 showing a protective effect and rs353300 an increased risk [50]. Extending this genetic insight, recent findings also indicate that specific CARMN SNPs, including rs13177623, rs11168100, rs12654195, and rs17796757, have been identified as key influencers of glioma risk and patient survival among the Chinese Han cohort [51]. Together, these findings underscore the significant impact of CARMN genetic variations across various diseases, highlighting their potential as invaluable diagnostic and prognostic tools in personalized medicine.

## 6. Regulatory Mechanisms of CARMN

The aberrant expression of CARMN is intricately linked to various human diseases, influenced by a range of upstream signaling molecules or stimulators. Factors identified to regulate CARMN expression include estrogen [15], TGF-β1 [13], PDGF-BB [19], hypoxia [52], β-elemene [26], doxorubicin (ADR) [24], and epigenetic modification [36]. Estrogen induces CARMN transcription by binding to the estrogen receptor β (ERβ) in the nuclei of BMSCs [15]. TGF-β1 elevates CARMN levels through Smad2/3 interaction with Smad-binding elements in the CARMN promoter region, facilitating transcriptional activation [13]. Conversely, PDGF-BB suppresses CARMN expression in serum-starved hCASMCs, reflecting its pivotal role in cellular response mechanisms [19]. Hypoxic conditions stimulate CARMN promoter activity, leading to increased transcription in human cardiomyocytes, which is critical under stress conditions [52]. β-elemene downregulates CARMN, thereby modulating the ILK/Akt pathway and influencing airway stenosis [26]. Doxorubicin exposure results in reduced CARMN and ERK5 expression in H9C2 cardiomyocytes, a cellular model for chronic heart failure, demonstrating the sensitivity of these genes to chemotherapeutic agents [24]. In CRC, m^6^A methylation suppresses CARMN expression by promoting its degradation, thereby facilitating tumor progression [36]. Additionally, as the host gene for the miR-143/145 cluster, the regulatory dynamics of CARMN are also pivotal in controlling the levels of miR-143/145 due to its sponge effects, thereby playing a significant role in gene silencing and post-transcriptional modulation [15]. This comprehensive understanding of CARMN regulation highlights its potential as a therapeutic target in disease treatment and prevention.

## 7. Conclusions

The lncRNA CARMN, serving as the host gene for the miR143/145 cluster, plays a crucial role in a wide array of physiological and pathophysiological contexts. It is essential for the differentiation of CPCs, maintenance of homeostasis in BMSCs and MaSCs, and the modulation of differentiation in SMCs and DPCs. In cardiovascular disease, CARMN is implicated in the development of atherosclerosis and abdominal aortic aneurysm by influencing VSMC plasticity and phenotype transformation. Additionally, CARMN functions as a tumor suppressor in cancers such as hepatocellular carcinoma, bladder cancer, and breast cancer, where its regulatory roles in cell invasion, migration, and proliferation highlight its potential as a beneficial biomarker and therapeutic target. In oncology, the suppression of tumor growth and metastasis by CARMN underscores its utility in clinical settings, providing a promising avenue for the development of cancer-specific therapies. Its interaction with transcriptional cofactors like MYOCD and SRF and its function as a miRNA sponge underscore its extensive role in gene regulation across both transcriptional and post-transcriptional processes.

Future research should focus on elucidating the specific roles of various CARMN isoforms in different tissues or cell types, particularly assessing their unique contributions to stem cells and SMCs. This detailed exploration is vital for a deeper understanding of their roles in cellular homeostasis, differentiation, and disease pathogenesis. Given its unique RNA properties and tissue-specific expression, CARMN presents a valuable biomarker for a diverse range of diseases. Advancing targeted therapies that modulate the expression or function of CARMN may pave the way for breakthrough treatments in both cardiovascular and oncological diseases, opening new paths in therapeutic development.

## Abbreviations

AAA	abdominal aortic aneurysm;
APC	adenomatous polyposis coli;
AS	atherosclerosis;
BLCA	bladder cancer;
BMSC	bone mesenchymal stem cells;
CARMN	cardiac mesoderm enhancer-associated non-coding RNA;
CC	cervical cancer;
CPC	cardiac precursor cell;
CRC	colorectal cancer;
EC	endometrial cancer;
GBM	glioblastoma;
GC	gastric cancer;
GI	gastrointestinal;
HCC	hepatocellular carcinoma;
LncRNA	long non-coding RNA;
LSCC	laryngeal squamous cell carcinoma;
SMC	smooth muscle cell;
VSMC	vascular smooth muscle cell.
